# Exploring Hydrogel Nanoparticle Systems for Enhanced Ocular Drug Delivery

**DOI:** 10.3390/gels10090589

**Published:** 2024-09-13

**Authors:** Zohreh Arabpour, Majid Salehi, Seungwon An, Amirhossein Moghtader, Khandaker N. Anwar, Seyed Mahbod Baharnoori, Rohan Jaimin Shah, Farshad Abedi, Ali R. Djalilian

**Affiliations:** 1 Department of Ophthalmology and Visual Science, University of Illinois, Chicago, IL 60612, USA; arabpour@uic.edu (Z.A.); moghtada@uic.edu (A.M.); kanwar@uic.edu (K.N.A.); roshku8@gmail.com (R.J.S.); fabedi3@uic.edu (F.A.); 2 Department of Tissue Engineering, School of Medicine, Shahroud University of Medical Sciences, Shahroud 3614773955, Iran; msalehi.te1392@gmail.com; 3 Clinical Stem Cell Laboratory, UI Blood & Marrow Transplant Program, University of Illinois Hospital and Health Sciences System, Chicago, IL 60612, USA; seunga1@uic.edu

**Keywords:** nanoparticle, hydrogel, ocular drug delivery, nanomedicine

## Abstract

Drug delivery to the ocular system is affected by anatomical factors like the corneal epithelium, blinking reflex, aqueous blood barrier, and retinal blood barrier, which lead to quick removal from the site and inefficient drug delivery. Developing a drug delivery mechanism that targets specific eye tissue is a major hurdle for researchers. Our study examines the challenges of drug absorption in these pathways. Hydrogels have been researched as a suitable delivery method to overcome some obstacles. These are developed alone or in conjunction with other technologies, such as nanoparticles. Many polymer hydrogel nanoparticle systems utilizing both natural and synthetic polymers have been created and investigated; each has pros and cons. The complex release mechanism of encapsulated agents from hydrogel nanoparticles depends on three key factors: hydrogel matrix swelling, drug-matrix chemical interactions, and drug diffusion. This mechanism exists regardless of the type of polymer. This study provides an overview of the classification of hydrogels, release mechanisms, and the role of controlled release systems in pharmaceutical applications. Additionally, it highlights the integration of nanotechnology in ocular disease therapy, focusing on different types of nanoparticles, including nanosuspensions, nanoemulsions, and pharmaceutical nanoparticles. Finally, the review discusses current commercial formulations for ocular drug delivery and recent advancements in non-invasive techniques. The objective is to present a comprehensive overview of the possibilities for enhancing ocular medication delivery through hydrogel nanoparticle systems.

## 1. Introduction

Research on ocular drug delivery devices has increased significantly in the last several years. Drug loss and low bioavailability, which are frequently encountered with conventional forms like eye drops, are two of the main obstacles to drug delivery to certain regions of the eye. These restrictions may result in insufficient therapeutic effects and the requirement for repeated dosage. Thus, a basic problem for pharmaceutical researchers is the creation of drug delivery systems that can deliver the medicine to the target site with minimal losses and systemic side effects [1].

Polymeric hydrogels, having unique properties, such as remaining in liquid form before administration and turning into gel because of exposure to body temperature or a change in pH and ionic conditions, have attracted considerable attention. Numerous hydrogels that react to changes in pH, temperature, or ions have been developed to extend the duration of contact with ocular tissues. These hydrogels present viable ways to use bioactive molecules to precisely time-regulate the distribution of both small- and large-molecular-weight medications [2].

One novel strategy for enhancing the delivery of medications to the eyes is nanotechnology. The goal of pharmaceutical nanotechnology is to produce medicinally effective substances in biocompatible nanostructures, including conjugates, micellar systems, nanoparticles, and nanocapsules. Numerous advantages are offered by these nanostructures, such as improved bioavailability, prolonged drug action in target tissues, targeted drug delivery, and increased stability against enzymatic or chemical degradation [3]. These benefits are supported by the intrinsic nanoscale size of these drug delivery systems, which is normally in the range of 10 to 1000 nm [4]. Drugs can be formed into nanoparticles to attach, dissolve, encapsulate, or trap them in a nanoparticle matrix. Nanoparticles can be created as nanospheres or nanocapsules, depending on the production technique used [5].

This paper explores the combination of hydrogel and nanoparticle systems for the transport of drugs to the eyes, highlighting the difficulties and most recent developments in this area. By exploring the potential of these innovative delivery systems, we aim to provide insights into their application in enhancing the therapeutic efficacy of ocular drugs.

## 2. Obstacles to Efficiently Delivering Medications to the Ocular System

Many obstacles that are inherent barriers and protective mechanisms prevent the effective delivery of drugs to the eye. The primary defense mechanism involves precorneal factors, which include the blink reflex, rapid tear reversal, and solution drainage through the tear ducts. Despite the potential storage capacity of about 30 μL in the ocular cavity, a significant fraction of administered eye drops are usually lost within 15–30 s of application [6].

The main obstacle for foreign materials trying to enter the eye is getting past many layers and into the target tissues, which include the cornea and conjunctiva. The epithelium, Bowman’s membrane, stroma, Dua’s layer, Descemet’s membrane, and endothelium are the six layers that make up the cornea, which is located in the anterior portion of the eye [7]. Its highly lipophilic nature hinders the entry of hydrophilic molecules, making it a significant barrier that restricts drug delivery [8].

**Figure 1:**
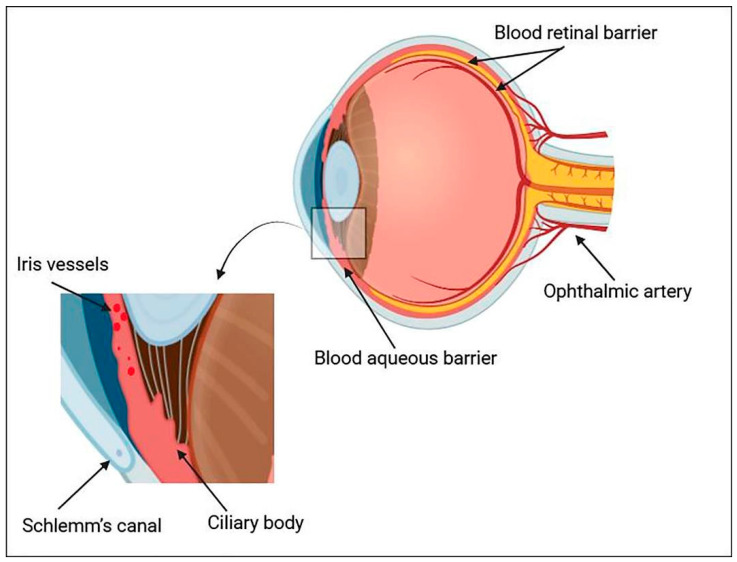
Depiction of the blood-aqueous barrier, which is composed of the ciliary body, Schlemm’s canal, and iris vessels, and the blood-retinal barrier, which is composed of the retinal pigment epithelium and the endothelial membrane of retinal vessels.

The conjunctiva, a highly vascular membrane covering the front of the eye, serves as an additional barrier. While the conjunctiva can help transport large, hydrophilic molecules, a significant portion of drugs bypass it and enter the bloodstream before reaching the eye. This adds to the topical medication’s ineffectual concentration in the back of the eye [9]. Aqueous blood barriers and retinal blood barriers are two more blood-ocular barriers that mostly keep chemicals from systemic circulation out of the eye. Drugs intended for the posterior region of the eye are frequently administered systemically; however, this method typically calls for high dosages, which may have serious side effects [10]. Figure 1 depicts these blood-ocular barriers and their respective tissues.

The goal of developing drug delivery systems is to increase the efficacy of the drug by increasing the active substance’s penetration into the eye and lengthening its residence duration at the application site. Adhesive biopolymers and hydrogel systems show potential in developing formulations with these vital benefits [11]. The increased viscosity of hydrogel systems also helps resist formulation clearance due to flashing and further increases bioavailability [12].

## 3. Hydrogels in Ocular Drug Delivery

Three-dimensional networks of polymers, known as hydrogels, are highly capable of absorbing water and biological fluids. The hydrogel polymer matrix contains hydrophilic groups, like –OH, –CONH–, –CONH2–, and –SO3H, which are responsible for this water-absorbing property. The content of the polymer and the kind of aqueous medium influence the degree of polymer hydration [13]. Hydrogels absorb a lot of water because of the important cross-links in their structure, but instead of dissolving in the surrounding aquatic environment, they swell. Polymer networks can form cross-links by physical entanglements, hydrogen bonds, covalent bonds, or van der Waals interactions. These cross-links can be broadly divided into two types: chemical and physical (such as entanglements or crystallites) [14].

### 3.1. Classification of Hydrogels

The synthesis and chemistry of the polymer network, quantitative material properties, interaction parameters, decay/release kinetics, and transport processes must all be understood to create a hydrogel system with predetermined physicochemical parameters and release profiles. The nature of side groups (neutral or ionic), mechanical and structural properties (affine or phantom), preparation technique (homopolymer or copolymer), physical structure (amorphous, semi-crystalline, hydrogen bonding, supramolecular, and hydrocolloid), and response to physiological stimuli (pH, ionic strength, temperature, electromagnetic radiation, etc.) are just a few of the characteristics that can be used to categorize hydrogels [15]. Polymers commonly used in the preparation of hydrogels for pharmaceutical and biological applications originate from natural or synthetic sources [16]. Natural hydrogels have a number of advantageous qualities, including non-toxicity and biocompatibility, despite the possibility that they have subpar mechanical qualities and that the presence of immunogenic or pathogenic moieties causes immunogenicity or triggers inflammatory reactions [16,17]. On the other hand, synthetic polymers, with their well-defined structures, can yield hydrogels with customizable degradation kinetics and mechanical properties [18]. While these materials are useful for a variety of applications in drug delivery systems, they also possess considerable physical and chemical features [18].

Ensuring adequate biodegradability and biocompatibility is crucial for hydrogels utilized in drug delivery and biomedical applications. To obtain the needed qualities, creative synthesis and cross-linking techniques are developed in response to this demand. Therefore, different cross-linking techniques are required to produce hydrogels appropriate for particular uses [19]. Furthermore, the monomers used to synthesize the hydrogel polymer networks have an impact on the properties and possible applications of hydrogels with various structures in addition to the preparation techniques [20].

### 3.2. Release Mechanisms from Hydrogel Matrices

The main method of drug release from hydrogels is passive diffusion, which permits molecules with different sizes and properties to readily enter and exit the hydrogel matrix at any time during the loading and storage stages [21]. Because of their hydrophilic qualities—particularly in terms of how they release incorporated chemicals—hydrogels differ from hydrophobic polymer matrices. Drug release techniques from hydrogels are classified into three groups: diffusion-controlled, swelling-controlled, and chemically regulated. This is the rate-limiting stage of the release phenomena. The release of active substances from hydrogel devices is controlled by these mechanisms. Drug diffusion out of the gel matrix is influenced by the mesh sizes of the matrix [22]. These mesh sizes are dependent on the degree of cross-linking, the chemical structure of the monomer, and, when possible, the type and potency of external stimuli. The mechanical strength, degradability, diffusion, and other physical characteristics of a hydrogel network are significantly influenced by the mesh size. Biomedical hydrogels frequently expand to mesh sizes between 5 and 100 nm, which are larger than most small molecule drugs’ mesh sizes [23]. Thus, unless the network structure and size of the swollen hydrogels are suitably designed to achieve optimal micromolar rates, the release of macromolecules such as oligonucleotides, peptides, and proteins exhibits sustained release, while the release of small molecule drugs is not significantly hindered in the swollen state. When a drug’s rate of release exceeds the hydrogel’s swelling, release due to the swelling control mechanism takes place, and the swelling controls the release behavior [24]. Chemical events taking place in the gel matrix, such as the breaking of polymer chains by hydrolytic or enzymatic degradation or the reversible/irreversible interactions between the released drug and the polymer network, further dictate chemically controlled release. In addition to these methods, the binding equilibrium of the drug-binding moieties included in the hydrogels and surface or bulk erosion of the hydrogels can also have an impact on drug release rates in certain circumstances [25].

### 3.3. Controlled-Release Hydrogel Systems

To address therapeutic needs, several different controlled release systems have been investigated to date. These systems regulate the release of medications or other chemicals in the body at a precise time or location in a predefined way. Hydrogels are the most suitable potential-controlled release systems going forward because of their special qualities. Delivery techniques based on hydrogels fall into two main categories: (1) systems that release the drug in response to stimulation and (2) time-controlled systems that release the drug gradually over time. Adaptations to changes in circumstances are planned based on physiological requirements at the appropriate time and location [26]. Despite considerable interest in stimuli-sensitive hydrogel-based drug delivery systems, stimuli-sensitive systems have slow response times. Although this can be solved by developing thinner and smaller hydrogels, it compromises the mechanical strength of the hydrogel polymer network [27].

Responsive hydrogels exhibit significant structural or functional changes in response to changes in their external environment. These hydrogel “smart” systems can be divided into three categories: (i) physical release-inducing systems, (ii) chemical release-inducing systems, and (iii) stimuli-inducing systems. Among the physical stimuli of interest are sound, sound pressure, temperature, electricity, light, and magnetic fields. Chemical stimuli include things like pH, solvent composition, ions, and specific molecular recognition events [28]. The capacity of temperature-sensitive hydrogels, in particular, to repeatedly experience swelling and dissolution in response to temperature changes has drawn attention and has shown promise for use in pharmacological applications [29]. Chemically responsive hydrogels, on the other hand, come in a variety of forms and release medication in reaction to variations in the concentration of particular molecules or bioactive substances within their surroundings [30].

## 4. Pharmaceutical Uses of Hydrogels

A great deal of research has been conducted on hydrogels to create the best possible drug delivery systems with desired therapeutic effects. Because of their unique physicochemical and biological properties, as well as their adaptability, they are considered to be very good candidates for the delivery of therapeutic drugs [31]. Various characteristics, such as the mode of administration, the type of material being administered, and the drug release kinetics, are used to categorize pharmaceutical hydrogels [32]. While it is uncommon to find a single classification scheme for therapeutic hydrogel formulations in the literature, grouping them according to the mode of administration allows for a wide range of these substances. Pharmaceutical hydrogels can, therefore, be divided into five categories. Hydrogel-based ocular delivery systems, topical and transdermal hydrogel systems, transdermal and implanted hydrogel systems, and oral hydrogel systems are among the hydrogel devices for gastrointestinal (GI) drug delivery. Additionally, hydrogel formulations have also been investigated for distribution via additional channels, like the nose and the vagina [33].

## 5. Hydrogel Nanoparticles

Hydrogel nanoparticles (NPs), commonly called nanogels, are the result of a substantial confluence of efforts in drug delivery research. These nanoscale particulate materials are interesting prospects for medicinal applications because they share traits and properties with both hydrogels and nanoparticles [34]. Hydrogel nanoparticles have been prepared using a variety of techniques, including the use of both naturally occurring hydrophilic polymers and synthetic polymers. The generated and characterized varieties of nanogels are described in the following sections, which are categorized based on the polymeric materials that were utilized to create them. For further technical information, readers are urged to consult original sources, even if this review covers a lot of important material [35].

### 5.1. Alginate

Alginic acid is an anionic biopolymer made up of linear chains of α-L-glucuronic acid and β-D-mannuronic acid. It has a number of noteworthy characteristics, such as being very soluble in water, having the capacity to create extremely porous gels under specific circumstances, being biocompatible, and not being harmful [36]. When counter-ions are introduced to alginate, the process of successive cross-linking and polymer network creation usually results in hydrogel-based drug delivery vehicles, such as microparticles and nanoparticles [36]. While this reaction can be started by a variety of cationic species, researchers frequently favor calcium chloride. Preparation methods are generally customized to control the gelation process, which affects the size range of the particles based on factors such as alginate concentration, counter-ion concentration, and the rate at which the counter-ion solution is added to the alginate solution [37]. To deliver daptomycin to the eye, for instance, Costa et al. [38] created chitosan-alginate-coated nanoparticles. After the medication was administered for four hours, these nanoparticles showed up to 92% encapsulation efficiency and 9–12% in vitro ocular permeability rates in retinal cell monolayers [38]. In another study, al-Juboori et al. [39] created a topical gel formulation using concentrations of 0.1%, 0.3%, and 0.5% sodium alginate along with ciprofloxacin and naproxen. This study showed that sodium alginate has temperature-dependent in situ gelling properties that lead to long-term persistence on the ocular surface.

### 5.2. Chitosan with Ionic Cross-links

The structural element of chitosan, a linear polysaccharide obtained from chitin, is present in arthropod exoskeletons. It is composed of repeated units of D-glucosamine and N-acetyl-D-glucosamine [40]. Particle-based medication delivery systems have been effectively developed by utilizing chitosan’s positive charge. Because of its cationic property, chitosan can form electrostatic complexes with a variety of biological fluids and membranes, medications, and anionic polysaccharides [41]. Chitosan undergoes a gelation transition at pH 6.5, resulting in deionization and the formation of a disordered three-dimensional structure. In addition to interacting with negatively charged polymers, chitosan also exhibits ionotropic gelation, forming gels upon contact with specific polyanions through cross-linking between and within polymer chains [41].

In recent studies, an ionotropic gel containing tripolyphosphate (TPP) was used to create chitosan nanoparticles for drug encapsulation. This method involves mixing an alkaline solution (pH 7–9) containing TPP with an acidic solution (pH 4–6) containing chitosan. The interaction between TPP phosphates and the amino groups of chitosan swiftly produces nanoparticles through intramolecular and intermolecular bonding [42]. A chitosan and dextran formulation was created by Chavan et al. [43] or ciprofloxacin ocular administration. With simulated tear fluid, this drug delivery system demonstrated a transport at pH 7.4 and sustained drug release over a 24 h period.

### 5.3. Polyvinyl Alcohol

A synthetic polymer with a linear structure, amphiphilic characteristics, semi-crystalline structure, biocompatibility, biodegradability, high flexibility, and non-toxicity is called polyvinyl alcohol, or PVA. By lowering the interfacial tensions in solutions, it functions as an emulsifier [44]. Vinyl acetate is polymerized by free radicals and hydrolyzed to form hydroxyl groups from acetate groups to produce PVA. This process results in a wide range of molecular weights that significantly affect the properties of the polymer, including crystallinity, adhesion, mechanical strength, and diffusion [45]. PVA has been recognized as a leading candidate for hydrogel research because of its versatile properties. Cross-linking of PVA chains can be achieved using physical methods (such as freezing and melting) as well as chemical methods (such as cross-linking agents, electron beams, or γ-irradiation). These cross-links are crucial for the application of PVA in various medical and pharmaceutical fields [46].

Bettina et al. [47] introduced a gentle, non-cryogenic method to create physical hydrogels using poly(ethylene glycol) to gel polyvinyl alcohol (PVA). They explored the impact of PVA molecular weight on the erosion behavior of these biomaterials. The study assessed these PVA hydrogels for their response in adherent macrophages, their ability to deliver cytotoxic drugs affecting myoblast proliferation, and their capacity to deliver growth factors promoting endothelial cell proliferation. Together, these results provide a thorough assessment of these adaptable matrices and demonstrate their potential use in tissue engineering and regulated drug delivery systems. [47]. To improve the loading and release of eye medicines, Xu et al. [48] developed hydrogels based on polyvinyl alcohol (PVA) and incorporating β-cyclodextrin (β-CD). This process creates methacrylate-β-CD (MA-β-CD) and methacrylate-PVA (PVAMA), which are further polymerized under UV light to produce pPVA-β-CD hydrogels. Purarin and acetazolamide, as pharmacological models, show improved loading and sustained release over a period of 15 days, indicating that β-CD is useful in lessening the drug’s early burst release. The previously described results highlight the potential of pPVA-β-CD hydrogels in the context of controlled administration of ocular medications [48].

### 5.4. Polyvinylpyrrolidone

The FDA has cleared polyvinylpyrrolidone (PVP), a hydrophilic polymer that was acceptable for Baharali et al. to develop a method to produce PVP-based hydrogel nanoparticles with diameters less than 100 nm by using the aqueous cores of reverse micellar droplets as nanoreactors [49]. PVP is highly valued for its outstanding characteristics, including biocompatibility, non-toxicity, pH stability, solubility in water and organic solvents, the ability to combine hydrophobic and hydrophilic substances, and chemical inertness in physiological reactions. These properties open numerous possibilities, such as the synthesis and stabilization of metal nanopowders, modification of various materials, advancement of modern technologies, and development of new functional materials. By changing the PVP’s molecular weight, the overall polymer matrix’s characteristics can be changed [50]. Drugs that are weakly soluble in water can form complexes with PVP, which greatly improves the solubility and stability of the former. By improving ocular permeability and therapeutic effects, Wang et al. employed these self-assembled complexes to investigate their potential as ocular drug delivery methods. In their study, naringenin (NAR) was delivered orally using PVP K-17PF-based nanocomplexes. The antioxidant activity and membrane penetration of NAR in vitro were significantly enhanced by the 17PF-NAR nanocomplexes. The ophthalmic solution was well tolerated in vivo in rabbits and showed good cellular tolerance in vitro. Furthermore, notable advancements in intraocular penetration and anti-inflammatory effectiveness were demonstrated by the 17PF-NAR nanocomplexes in vivo [51].

### 5.5. Polyethylene Oxide (PEO) and Poly Ethyleneimine (PEI)

A kind of nanomaterial, PEO-cl-PEI, blends cross-linked polyethylene oxide (PEO) and polyethyleneimine (PEI), forming dispersed networks that interact with anionic or amphiphilic molecules and oligonucleotides to create stable nanocomposites. These materials combine hydrophobic regions linked by hydrophilic PEO chains, maintaining stable aqueous dispersions despite the collapse of gel particles because of polyion complex formation [3]. They make it easier for physiologically active substances, including retinoic acid, indomethacin, oligonucleotides, and hydrophobic molecules, to be effectively immobilized. Redesigned nanogel particles demonstrate effective cellular absorption and intracellular release of oligonucleotides. These particles are fitted with polypeptide ligands to improve receptor-mediated delivery. The reaction between the amino groups of PEI and functionalized PEO forms transparent hydrogels quickly in homogenous aqueous solutions in 3–5 min, retaining around 50 times their dry weight in water. A minimum PEO/PEI molar ratio of six or greater can be used to produce hard hydrogels. A modified solvent emulsification/evaporation process is used to create finely dispersed systems, and gel permeation chromatography is then used to separate the nanogel particles [52].

PolyGelTM, an in situ gel system made of poly(α-carboxylate-co-α-benzyl carboxylate-ε-caprolactone)-block-poly(ethylene glycol)-block-poly(α-carboxylate-co-α-benzyl carboxylate), was investigated by Alshamsan et al. [53] for its effectiveness in delivering cyclosporine A (CyA) in the treatment of uveitis. They contrasted PolyGelTM with non-gelling micelle formulation PEO-b-PCL and Restasis^®^, which are produced by AbbVie company in the USA. Through stimulation trials, this study assessed the drug’s ocular tolerance and bioavailability. It found that, although PolyGel^TM^ and PEO-b-PCL were well tolerated, Restasis^®^ had the highest ocular bioavailability of CyA. The longest length of medication penetration into the eye was demonstrated by PolyGel [53].

### 5.6. Poly-N-Isopropylacrylamide

PNIPAM, often known as poly-N-isopropylacrylamide, is a highly recognized type of responsive polymer. When PNIPAM chains dissolve in water, they show a low critical solution temperature. This significant change is thought to be caused by the dissolution of hydrogen bonds between water molecules that surround the amide groups on the side chains of the polymer [54]. Ross et al. [55] developed chitosan-linked poly(N-isopropylacrylamide)-based thermogels to provide sustained release of ketotifen fumarate for treating allergic conjunctivitis, aiming to replace traditional eye drops. They modified the base polymer thermogel properties by incorporating hydrophilic (acrylic acid) and hydrophobic (methyl methacrylate) comonomers, which were cross-linked with chitosan. The thermogels had a lower critical solution temperature below the eye surface temperature and exhibited over 80% equilibrium water content post-gelation. By altering the procedure and chitosan content, the researchers were able to change the material’s rheological qualities and the rate at which ketotifen fumarate was released, ranging from 40% to 60%. Studies conducted in vivo and in vitro verified that thermogels are non-toxic. When applied to the inferior fornix of the eye, ketotifen fumarate was continuously released over a number of days, suggesting a potential substitute for traditional eye drops in the management of allergic conjunctivitis [55].

## 6. Nanotechnology in Ocular Disease Therapy

In order to alter structures and properties at the nanoscale, or more precisely between 1 and 100 nm, nanotechnology combines science and technology [56]. The field of “nanomedicine”, which includes basic science, clinical therapy, diagnostics, and disease management, benefits from this exact control at the molecular level. Nanotechnology has been used in ocular disease medicine delivery systems since the 1980s [57]. Nano formulations can overcome ocular barriers, enhance drug residence time on the corneal surface, improve permeability and bioavailability, reduce the degradation of unstable drugs, and offer better patient tolerance compared to conventional drugs. Organic and inorganic nanoparticles (NPs) offer new solutions to address unmet clinical needs in ophthalmology and greatly enhance drug delivery. Various formulations of nanoparticles have been developed, including lipid-based nanoparticles, nanosuspensions, and nanoemulsions [58].

### 6.1. Lipid-Based Nanoparticles

Lipids are water-insoluble organic compounds characterized by molecules with polar hydrophilic heads and non-polar hydrophobic tails. While normally hydrophobic, some lipids have amphipathic properties.

Their distinctive properties, including crystallinity, specific melting points, and polymorphic behavior, make lipids very effective as carriers for ocular drug delivery [59]. The use of lipid-based nanocarriers in ocular drug delivery offers several advantages, including formulation flexibility, improved bioavailability, solubility, and optimal penetration with minimal risks. In addition, these nanocarriers allow for more controlled release of ophthalmic drugs and enhance therapeutic outcomes [60]. For instance, in both preclinical and clinical investigations, topical liposome-based nanosystems have demonstrated effectiveness in delivering triamcinolone acetonide (TA) to the vitreous and retina. This development in ocular pharmacology may reduce or eliminate the necessity for intravitreal injections in the treatment of a number of intraocular inflammatory and neovascular illnesses [61]. Although injecting TA intravitreal is a well-established and efficient method of administering this steroid to the back of the eye, there is a chance of major side effects, including endophthalmitis, injury to the lens, and retinal detachment. These risks can be avoided by using liposome-based topical nanosystems. In addition, topical use allows immediate cessation of steroid effects upon discontinuation of use, whereas intravitreal injection effects persist for a long time until the drug wears off. This makes the management of side effects in intravitreal injection patients more challenging [60]. For the treatment of ocular inflammatory disorders, steroidal and non-steroidal anti-inflammatory medications have been delivered via lipid-based nanosystems. Many anti-inflammatory medications with different molecular architectures are now being investigated. Examples include nanostructured lipid carriers (NLCs) holding ibuprofen or flurbiprofen, solid lipid nanoparticles (SLNs) encapsulating diclofenac sodium, and SLNs containing cyclosporine A for severe ocular inflammation [62]. A preference for simpler nanoparticles, such as liposomes and micelles, for drug delivery is evident. In addition, research has shown the frequent use of polymers as vehicles to improve the physicochemical properties of formulations and enhance ocular drug delivery [63].

### 6.2. Nano Suspensions

The pharmaceutical industry faces two primary issues in medication formulation: poor solubility and low bioavailability. Newly developed medicines have low water solubility. Although there are several approaches used to improve medication solubility, each has drawbacks. As a result, researchers have discovered that using nanosuspensions to administer drugs is another effective strategy [64]. Nanosuspensions are nanoparticles with colloidal dispersions stabilized by surfactants. One of the most important advantages of nanosuspensions is the continuous release of poorly soluble drugs on the surface of the eye, which leads to an increase in shelf life [65]. Nanosuspensions have been used in oral and topical formulations to improve the bioavailability of ophthalmic drugs. Ibuprofen sodium salt nanosuspension (IBU) coated with Eudragit RS 100 polymer which manufactured by Evonik Industries in Germany, as well as glucocorticoids such as dexamethasone, hydrocortisone, and prednisolone formulated in nanosuspensions for the treatment of eye inflammation, have shown long-term drug absorption and higher bioavailability in ocular drug delivery, and as a result, they reduce the frequency of administration [66]. Formica et al. designed a study to increase the therapeutic efficacy of triamcinolone acetonide (TA) for eye inflammatory disorders by creating nanocrystals (TA-NC). In vivo evaluation in a rabbit model of endotoxin-induced uveitis (EIU) showed that transconjunctival administration of TA-NC effectively reduced inflammation in the anterior chamber and iris without causing ocular damage [67].

### 6.3. Nano Emulsions

Colloidal systems known as nanoemulsions are made up of two immiscible liquid phases, either water in oil or oil in water, with droplet sizes varying between 20 and 200 nm. By interacting with the lipid layer of the tear film, nanoemulsions improve the solubility and bioavailability of ocular medicines. They also offer a steady and reliable delivery of medicine and lessen the need for frequent therapeutic dosages. Notably, because these formulations can deliver both hydrophilic and lipophilic medicines, they can take the place of liposomes and traditional ophthalmic formulae [60].

A study by Ammar et al. [68] on the anti-glaucoma drug dorzolamide hydrochloride in the form of nanoemulsion showed long-term effects, a rapid onset of effects, and a reduced need for frequent administration of eye drops. Prajapati et al. [69] effectively formulated a sustained-release brimonidine tartrate formulation using an in situ self-nanoemulsion gel, which is non-irritating. Improving drug delivery to the posterior part of the eye increases the potential for the effectiveness of glaucoma treatment. Thiel et al. [70] created terbinafine hydrochloride nanoemulsion gels, which may extend the medication’s shelf life and improve its absorption. Mahbubian et al.‘s clinical study [71] assessed the absorption of acyclovir nanoemulsion in bovine corneas, demonstrating the formulation’s safety for treating ocular infections and increasing corneal penetration without irritating rabbit eyes. Another illustration is the application of dorzolamide hydrochloride nanoemulsions, which, when applied topically, exhibit therapeutic effects with a prompt and stable effect and successfully lower intraocular pressure [71].

## 7. Current Commercial Formulations for Ocular Drug Delivery

Various formulations are available to treat eye diseases, but each has its own challenges in delivering the drug effectively. Eye drops, the most widely used treatment method, exist in two types of solution and suspension. These conventional eye drops often require repeated use, sometimes several times a day, especially for chronic conditions like glaucoma, which can lead to unwanted side effects [72]. This circumstance emphasizes the necessity for cutting-edge drug delivery technologies, like hydrogels, which can gradually increase patient adherence, minimize adverse effects, and decrease the frequency of doses [73]. Although ointments have the advantage of reducing rapid drainage due to their higher viscosity compared to liquid formulations, they may cause blurred vision after application and potentially hinder patient compliance [73].

Subtenon, intravitreal, or systemic procedures are frequently used as treatment alternatives for disorders affecting the posterior region of the eye, each with its own set of disadvantages. Reducing the level of invasiveness of current medicines is the aim of developing new drug delivery strategies for these conditions. For example, antivascular endothelial growth factors, or anti-VEGFs, are commonly used to treat a number of posterior segment issues, particularly those that cause retinal damage, such as myopic choroidal neovascularization and diabetic macular edema. However, the large and hydrophilic nature of anti-VEGF molecules prevents them from passing past ocular barriers, necessitating intravitreal injections. This highlights the need to create state-of-the-art delivery systems and technologies that can administer drugs like anti-VEGF without necessitating regular, invasive injections [74].

Corticosteroids are approved for treating eye inflammation and pain post-surgery but are limited in long-term use because of risks like increased intraocular pressure (IOP) and cataract formation. Precise targeting is essential to avoid impacting surrounding tissues, while the corneal barrier and tear drainage hinder effective drug penetration and retention [75]. Safety requires non-toxic and irritation-free formulations, and sustained release systems are needed to reduce dosing frequency. Tailored treatments are necessary because of patient-specific factors. Advances such as nanoparticles, innovative devices, enhanced penetration techniques, personalized medicine, biocompatible materials, and patient-centered designs are crucial to improving efficacy and adherence in managing postoperative ocular inflammation. At present, several commercial implants, including Ozurdex and Dextenza, are available for treating ocular diseases [76].

One major challenge with corneal transplants is immune rejection, which often leads to transplant failure. To address this, immunosuppressants like rapamycin (RAPA) and antibiotics such as levofloxacin hydrochloride (Lev) are typically used post-transplant. However, these medications suffer from low ocular bioavailability and can lead to significant side effects [77]. Lev@RAPA micelles, which are a combination of levofloxacin and rapamycin encapsulated in micelles for potential ophthalmic applications, are still under development and not yet commercially available on the market. These hydrogels potentially reduce the risk of corneal graft rejection in corneal transplantation [77]. Some commercial hydrogel-based products available in the market for ophthalmic drugs are presented in Table 1 and Table S1.

## 8. Conclusions

This review underscores the ongoing challenges in delivering therapeutic agents to the eye and emphasizes the potential of hydrogel nanoparticle systems to address these issues effectively. By exploiting the unique properties of hydrogels and nanoparticles, researchers have made significant strides in increasing drug bioavailability, prolonging drug activity, and minimizing systemic side effects. The incorporation of nanotechnology offers viable solutions for targeted drug delivery to ocular tissues that address critical issues, such as rapid clearance and limited absorption associated with traditional delivery methods. As research advances in this interdisciplinary field, further innovations in formulation strategies and understanding of release mechanisms will undoubtedly pave the way for more effective ophthalmic drug therapies, ultimately benefiting eye disease patients worldwide.

## Figures and Tables

**Table 1:** Commercially hydrogel matrix drugs with nanoparticles for ocular drug delivery.

Commercial Name	Company	Hydrogel Matrix	Nanoparticles	Therapeutic Indication	Ref.
Dextenza	Ocular Bedford, USA, Therapeutix	Polyethylene glycol (PEG)	Dexamethasone	Post-surgical inflammation	[78]
Iluvien	Alpharetta, USA, Alimera Sciences	Polyvinyl alcohol (PVA) membrane	Fluocinolone Acetonide	Diabetic macular edema	[79]
Durysta	North Chicago, USA, Allergan	Polylactic acid and glycolic acid (PLGA)	Bimatoprost	Open-angle glaucoma and ocular hypertension.	[80]
Retisert	Rochester, USA, Bausch & Lomb	PVA	Fluocinolone Acetonide	Chronic noninfectious uveitis.	[81]
Ozurdex	North Chicago, USA, Allergan	PLGA	Dexamethasone	Macular edema and uveitis	[57]
Verisome	Newark, USA, Icon Bioscience Inc	PLGA	Dexamethasone	Macular edema	[82]
Ocusert	Vacaville, USA, Ocusert	Ethylene vinyl acetate (EVA)	Pilocarpine	Glaucoma	[83]

## Data Availability

Not applicable.

## Supplementary Materials

The following supporting information can be downloaded at: https://www.mdpi.com/article/10.3390/gels10090589/s1, Table S1: Nanoparticle-based research for ocular drug delivery.
