# Supplementary material for: Exploring Hydrogel Nanoparticle Systems for Enhanced Ocular Drug Delivery
Source: Gels. 2024 Sep 13;10(9):589. doi: 10.3390/gels10090589 (PMC11430953; doi:10.3390/gels10090589)
Supplement: Supplementary file 1 [file gels-10-00589-s001.zip › gels-3163597-supplementary.pdf]

TableS1: Nanoparticle-based research for ocular drug delivery

Polymer/Lipid Type	Drug	Therapeutic Indication	Ref.
Alginate Nanoparticles	Timolol	Enhanced corneal penetration; sustained drug release	[69]
Chitosan Nanoparticles	Cyclosporine A	Increased drug bioavailability; reduced inflammation	[70]
Gelatin Nanoparticles	Dexamethasone	Prolonged drug release; reduced ocular inflammation	[71]
Polyacrylamide Nanoparticles	Brimonidine	Improved drug penetration; sustained release	[72]
Poly(lactic-co-glycolic acid) (PLGA) Nanoparticles	Bevacizumab	Prolonged release up to 50 days; effective Vascular endothelial growth factor (VEGF) inhibition	[64]
Poly(ε-caprolactone) (PCL)	Dexamethasone	Sustained drug release; reduced intraocular pressure	[73]
Liposome Nanoparticles	Doxorubicin	Improved drug retention; reduced systemic toxicity	[74]
Solid Lipid Nanoparticles (SLN)	Tobramycin	Enhanced corneal penetration; prolonged drug release	[75]
Nanoemulsions (Lipid-Based)	Brinzolamide	Improved drug stability; enhanced corneal permeation	[76]
Nanosuspensions (Lipid-Based)	Besifloxacin	Anti-ocular bacterial infection	[77]
